# African Swine Fever Virus Circulation between Tanzania and Neighboring Countries: A Systematic Review and Meta-Analysis

**DOI:** 10.3390/v13020306

**Published:** 2021-02-15

**Authors:** Jean N. Hakizimana, Clara Yona, Olivier Kamana, Hans Nauwynck, Gerald Misinzo

**Affiliations:** 1 SACIDS Africa Centre of Excellence for Infectious Diseases, SACIDS Foundation for One Health, Sokoine University of Agriculture, P.O. Box 3019 Morogoro, Tanzania; clara.yona@sacids.org; 2 Department of Veterinary Microbiology, Parasitology and Biotechnology, College of Veterinary Medicine and Biomedical Sciences, Sokoine University of Agriculture, P.O. Box 3019 Morogoro, Tanzania; 3 Department of Biosciences, Solomon Mahlangu College of Science and Education, Sokoine University of Agriculture, P.O. Box 3019 Morogoro, Tanzania; 4 Department of Applied Research and Development and Foresight Incubation, National Industrial Research and Development Agency, P.O. Box 273 Kigali, Rwanda; olivier.kamana@nirda.gov.rw; 5 Laboratory of Virology, Faculty of Veterinary Medicine, Ghent University, Salisburylaan 133, 9820 Merelbeke, Belgium; Hans.Nauwynck@ugent.be

**Keywords:** African swine fever virus, phylogeography, ancestral character reconstruction, Eastern Africa

## Abstract

For over 100 years after the description of the first case of African swine fever (ASF) in Kenya, ASF virus (ASFV) cross-border spread in eastern and southern Africa has not been fully investigated. In this manuscript, we reviewed systematically the available literature on molecular epidemiology of ASF in Tanzania and its eight neighboring countries in order to establish the transmission dynamics of ASFV between these countries. Data were retrieved from World Animal Health Information System (WAHIS), Google Scholar, PubMed, Scopus, and CrossRef databases, using the recommendations of the Preferred Reporting Items for Systematic Reviews and Meta-Analyses (PRISMA) guidelines and reviewed to document ASF outbreaks and ASFV genotypes distribution. Using phylogeographic approach applied to ASFV p72 sequence dataset, the evolutionary history and the dispersal pattern of the ASFV strains were assessed. From 2005 to 2019, a total of 1588 ASF outbreaks affecting 341,742 cases that led to 302,739 domestic pig deaths were reported. The case fatality rates (CFR) varied from 15.41% to 98.95% with an overall CFR of 88.58%. Fifteen different p72 ASFV genotypes were reported and the time to the most recent common ancestor (TMRCA) for ASFV strains dated back to 1652.233 (1626.473, 1667.735) with an evolutionary rate of 4.805 × 10^−5^ (2.5857 × 10^−5^, 9.7789 × 10^−5^). Phylogeographic dispersal analysis revealed several transboundary spread events of ASFV strains between these countries. These results suggest persistent circulation of ASFV in these countries and advocate for more research to improve our understanding of the transmission dynamics of the virus and for a regional approach to mitigate the spread of ASFV.

## 1. Introduction

African swine fever (ASF) threatens global domestic pig industry and wild boars due to its high mortality rate, trade restriction [1] and hinders poverty alleviation, an important component of Sustainable Development Goals of the United Nations in affected countries. African swine fever was first described in Kenya in 1921 [2] and since then it has become endemic in many countries of Africa, South of the Sahara where it threatens food security and livelihoods of poor and marginalized communities keeping domestic pigs for subsistence [3,4,5]. The first wave of ASF outside Africa started in Portugal in 1957 and in 1960 with subsequent spread to Iberian Peninsula, other countries of the European Union, the Caribbean and Brazil before its eradication in these countries in 1990 with the exception of Sardinia, Italy where it remains endemic since 1978 [6,7,8]. The second wave of ASF in Europe started with an introduction of the virus in Georgia in 2007 and from there, ASF expanded its geographical distribution to Caucasus region, European Union countries, and later on in August 2018 to Asian countries including China [1,9,10,11]. The expanding geographical distribution of ASF poses a threat to ASF-free countries worldwide and the maintenance of ASF in countries where domestic pigs are kept for subsistence including eastern and southern African countries is believed to fuel the global spread and risk of ASF [1,12]. For instance, the first spread of ASF virus (ASFV) genotype I to Portugal in 1957 was suspected to come from Angola while Madagascar is speculated to be the origin of the ASFV genotype II introduction into Georgia in 2007 [8,13].

African swine fever is caused by the ASF virus (ASFV), a linear double-stranded DNA arbovirus with an icosahedral morphology that was once included in the family of *Iridoviridae*, but is now assigned to the genus *Asfivirus*, family *Asfarviridae* [14], being the only member of this family and the only known DNA arbovirus. Depending on the viral isolate, the ASFV genomes vary in length from about 170 to 193 kilobase pairs and encode between 151 and 167 open reading frames with a conserved central region of about 125 kbp and variable termini [15]. These variable ends encode five multigene families (MGF) that contribute to the variability of the virus genome. Molecular characterization of distinct genome regions of ASFV has proved to be very useful in elucidating the origin and transmission pathways of ASF during outbreaks [16,17]. Different genomic regions have been targeted to detect ASFV phylogenetic relationships with different levels of precision [16]. The current approach for investigating the molecular epidemiology of ASF is through sequencing of the C-terminal end of p72 (*B646L*) gene encoding the p72 major capsid protein in order to determine the viral genotype [18]. So far, 24 ASFV p72 genotypes (I–XXIV) have been identified [19,20]. Further discrimination into subgroups of closely related viruses is usually conducted by sequence analysis of the tandem repeat sequences (TRS) located in the central variable region (CVR) within the *B602L* gene [21,22,23] and the intergenic region between the *I73R* and *I329L* genes [24,25]. Several other genomic regions such as the *E183L* encoding the p54 protein, the *CP204L* encoding the p30 protein, and the *EP402R* gene encoding the CD2v protein, have proved to be useful tools for molecular epidemiological and virus spread investigations [13,21,26].

Four transmission cycles have been described for ASFV: (1) a sylvatic cycle where the virus asymptomatically circulates between wild suids (mainly warthogs, *Phacochoerus africanus*) and soft ticks of the *Ornithodoros moubata* complex inhabiting warthog burrows, (2) a tick to domestic pig cycle characterized by the transmission of the virus to domestic pigs by ticks dropped to domestic pig shelters by warthogs, (3) a domestic cycle accounting for most of ASF outbreaks globally where the virus is transmitted by direct contact between infected and susceptible domestic pigs or from infected domestic pig products to domestic pigs, and (4) a wild boar-habitat cycle specific to Eurasian countries [5,8,27]. The sylvatic ASFV cycle specific to eastern and southern Africa is believed to play an important role in the genetic variation of the ASFV and in hindering ASF control [20]. African swine fever outbreaks in domestic pigs are mandated to be reported to the World Organization for Animal Health (OIE) and despite the perceived increase of the reported ASF outbreaks [10], our understanding of the process of ASFV maintenance and spread in eastern and southern Africa is quite limited. The aim of this manuscript was to review systematically the available literature on molecular epidemiology of ASF in Tanzania and neighboring countries in order to establish the transmission dynamics of ASFV between these countries. This information will inform the effective application of ASF control measures and will highlight research gaps warranted for further investigation.

## 2. Materials and Methods

### 2.1. Search Strategy

The African swine fever outbreaks records were retrieved from OIE, World Animal Health Information System (WAHIS). Using the recommendations of the Preferred Reporting Items for Systematic Reviews and Meta-Analyses (PRISMA) guidelines [28], a literature search was carried out at Google Scholar, PubMed, Scopus, and CrossRef databases to find relevant information related to molecular epidemiology of ASF in Tanzania and its neighboring countries. The following search string was used: “African swine fever” OR “African swine fever virus” OR “ASF” OR “ASFV” AND (“Tanzania” OR “Burundi” OR “Democratic Republic of the Congo” OR “Kenya” OR “Malawi” OR “Mozambique” OR “Rwanda” OR “Uganda” OR “Zambia”). The search did not include any limit regarding publication date and articles in English were included in the review. Rayyan QCRI [29] was used for preliminary screening of studies. Articles from PubMed were imported directly to Rayyan QCRI, while the Publish or Perish Software [30] was used to import articles from other databases. A first screening of titles and abstracts was performed, then full text of articles identified as possibly relevant were reviewed. The bibliographies of included articles were assessed for further eligible publications.

### 2.2. Inclusion and Exclusion Criteria

Two independent reviewers (JNH and CY) electronically searched for studies and screened them according to eligibility criteria, the last search was done on 14th October 2020. The focus was on the articles published in English that describe molecular epidemiology of ASFV in Burundi, Democratic Republic of the Congo (DRC), Kenya, Malawi, Mozambique, Rwanda, Tanzania, Uganda, and Zambia without limit regarding publication date. Abstracts without available full text, articles in language other than English, review articles, duplicated information, and studies describing only ASF diagnosis without sequencing and phylogenetic analysis in order to identify the ASFV genotype were excluded.

### 2.3. Data Extraction and Analysis

Relevant data from all articles included in the review were extracted and entered in a Microsoft Excel database for further handling. The following data were summarized: authors’ name, year of publication, host species, nature of sample (blood, tissue, etc.), genes sequenced, sequences accession numbers, country of the study setting, town or the district of the study (where available), and summary of the findings. Primary authors were contacted in case some data were missing or unclear. The results were described using figures and tables to depict the trend of ASF occurrence over time and space.

### 2.4. Phylogeographic Analysis

In total, 126 ASFV p72 nucleotide sequences were downloaded from the NCBI GenBank database comprising ASFV strains from Burundi (*n* = 3), DRC (*n* = 22), Kenya (*n* = 17), Malawi (*n* = 17), Mozambique (*n* = 14), Tanzania (*n* = 15), Uganda (*n* = 11), and Zambia (*n* = 27). No sequences were found from Rwanda. The included sequences were collected from 1954 to 2019 and they were aligned by CrustalW using MEGA X [31] and subsequently edited using SeaView version 4 [32]. The resulting alignment consisted of 126 sequences (409 nucleotides long). The Smart Model Selection in PhyML (SMS) version 1.8.4 was used to evaluate the best fitting nucleotide substitution model using Akaike information criterion (AIC) as a model selection criterion [33]. The best fitting model for sequences used in this study was the GTR + G (general time reversible model with gamma distributed among-site rate heterogeneity). Therefore, a maximum likelihood phylogenetic tree was reconstructed under the GTR + G model with a bootstrap frequency of 1000 replicates as implemented by MEGA X [31]. TempEst version 1.5.3 [34] was used for temporal signal investigation in the dated-tip tree for further phylogenetic molecular clock analysis. The sequence data were annotated with year of the collection as stated in the corresponding original paper and the country where samples were collected. The least square dating (LSD) was used for ancestral events dating and rooting the phylogenetic tree based on dates [35] using strict molecular clock. The phylogeography of ASFV was reconstructed from the time-scaled tree generated by LSD and location annotations using PastML with maximum likelihood marginal posterior probabilities approximation (MPPA) and Felsenstein 1981 (F81) model options [36]. The PastML generated tree was visualized and edited using iTOL [37].

## 3. Results

### 3.1. Article Selection

**Figure 1:**
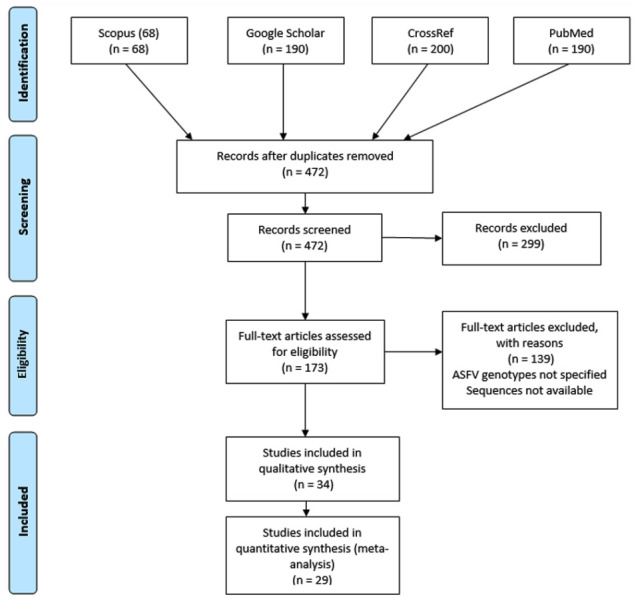
Preferred Reporting Items for Systematic Reviews and Meta-Analyses (PRISMA) flow chart of the literature search, screening, assessing eligibility, and article selection.

In total, 648 articles were collected during the initial search and 472 were included after elimination of duplicates. After titles and abstracts screening against the eligibility criteria, 299 articles were excluded. The majority of excluded publications reported data from countries not concerned by this review and review papers. Therefore, 173 full-text manuscripts were assessed in detail and 34 articles were retained for qualitative synthesis and 29 papers for further molecular assessment (Figure 1). The earliest study meeting the eligibility criteria was Lubisi et al. published in 2005, and therefore the current review considers literature over a 15 years period from 2005 to 2020.

### 3.2. ASF Disease Pattern

The total of ASF outbreaks reported to OIE by Tanzania and its eight neighboring countries from 2005 to 2019 were 1588 affecting 341,742 cases that led to 302,739 domestic pig deaths during this period. The case fatality rates (CFR) varied from 15.41% to 98.95% with an overall CFR of 88.58%. The number of reported ASF outbreaks, number of cases, number of deaths, and the case fatality rate reported by Tanzania and its eight neighboring countries increased over time from 2005–2009 to 2010–2014 periods (Table 1).

### 3.3. ASFV Genotypes

**Figure 2:**
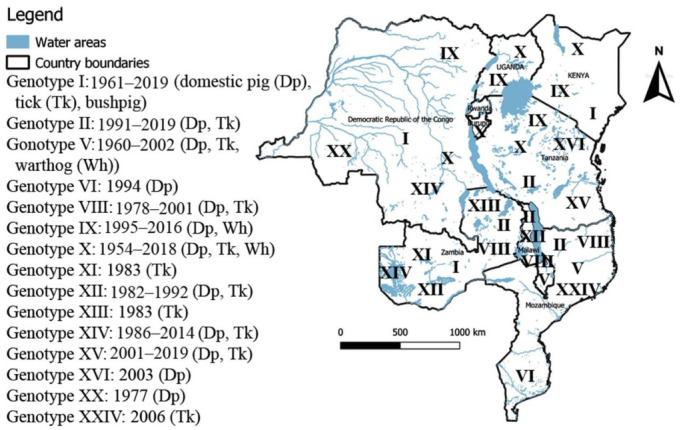
Distribution of the ASFV p72 genotypes circulating between Tanzania and its eight neighboring countries by October 2020. The map was developed using QGIS version 3.4.4 (https://www.qgis.org/en/site/about/index.html).

The included studies reported 15 different p72 ASFV genotypes in Tanzania and its eight neighboring countries. The following genotypes were reported: I, II, V, VI, VIII, IX, X, XI, XII, XIII, XIV, XV, XVI, XX, and XXIV (Table 2 and Table 3). Genotypes V and X were found in domestic pigs, warthogs and ticks; genotype I in domestic pig, ticks and bush pig; genotype IX in domestic pigs and warthogs; genotypes II, VIII, XII, XV, and XIV in both domestic pigs and ticks; while genotypes VI, and XVI were exclusively described in domestic pigs, and genotypes XI, XIII, and XXIV exclusively in soft ticks (Figure 2). Zambia recorded a wider variety of ASFV p72 genotypes with seven genotypes being reported to circulate in the country, namely genotypes I, II, VIII, XI, XII, XIII, and XIV.

### 3.4. Phylogeography of ASFV

**Figure 3:**
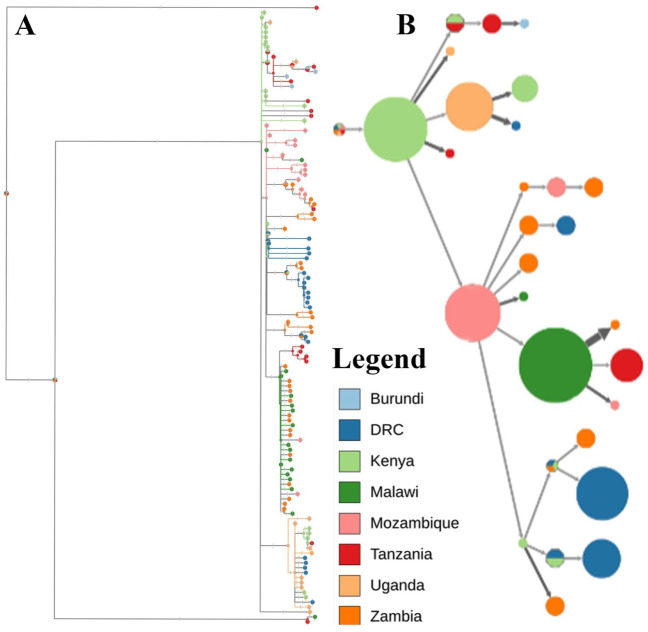
Ancestral reconstruction of African swine fever virus strains collected in Tanzania and its neighboring countries. The figure shows the full tree (**A**) and compressed (**B**) visualizations produced by PastML using MPPA with an F81-like model. Different colors correspond to different countries as shown in the legend.

Results for root to tip divergence showed that the dataset used in this study had a positive temporal signal with the correlation coefficient of 0.19 and R^2^ of 0.038. The time to the most recent common ancestor (TMRCA) for ASFV strains circulating between Tanzania and its neighboring countries dated back to 1652.233 (1626.473, 1667.735) with an evolutionary rate of 4.805 × 10^−5^ (2.5857 × 10^−5^, 9.7789 × 10^−5^). The location of the root was unresolved with an indication that Kenya might be the root location with 14.95% probability. Phylogeographic dispersal of ASFV revealed several transboundary spread events from Kenya to Uganda, Tanzania, and Mozambique. From Mozambique, ASFV further spread to Zambia and Malawi and from there to Tanzania. Transmissions from Uganda to DRC, from Tanzania to Burundi were also observed (Figure 3).

## 4. Discussion

This review aimed at investigating the molecular epidemiology of the ASFV strains circulating in Burundi, DRC, Kenya, Malawi, Mozambique, Rwanda, Tanzania, Uganda, and Zambia. Data and articles describing molecular epidemiology of ASF were retrieved from public databases and 34 articles met our inclusion criteria. Our findings suggested a tendency toward the increase of number of reported ASF outbreaks, number of cases, number of deaths, and the case fatality rate over time. A high ASFV genotypic diversity was reported by the included studies with 15 different ASFV genotypes. Additionally, phylogeographic dispersal analysis revealed several transboundary spread events of ASFV strains between countries concerned by this review.

All nine countries concerned by this review reported 1588 ASF outbreaks to the OIE WAHIS from 2005 to 2019 [10]. The real number of ASF outbreaks that occurred in these countries during the same period may be higher than those reported to OIE because the ASF outbreaks reporting in Africa was characterized as sporadic and incomplete for several reasons including poor communication channels and fear of undesirable consequences [4,5]. The increasing trend of reported ASF outbreaks, cases and deaths of domestic pigs could be attributed to the increasing number of ASF outbreaks within the region, with DRC and Malawi reporting 153,692 and 80,437 ASF cases that led to 140,493 and 77,896 domestic pig deaths, respectively, between 2010 and 2014 [10]. In addition, there has been an improvement in laboratory diagnosis capacity and enhanced surveillance systems in order to prevent ASF outbreaks in concerned countries. However, this increase of reported ASF outbreaks is worrisome and regional control programs need to be put in place to control the occurrence of ASF in the region. For some countries (Burundi, Kenya, Malawi, Rwanda, and Zambia), the data for 2019 are not complete because at the time of data retrieving, their 2019 reports were not available on the WAHIS database.

This review showed that 15 of the 24 ASFV p72 genotypes have occurred in Burundi, DRC, Kenya, Malawi, Mozambique, Tanzania, Uganda, and Zambia. These countries are rich in wildlife protected areas where ASFV natural reservoirs exist, making the epidemiology of the ASFV in these countries complex. For instance, the included studies reported diverse range of ASFV p72 genotypes recovered from domestic pigs, warthogs, ticks, and bush pigs in samples collected from 1954 to 2019. Genotypes V and X were isolated from domestic pigs, warthogs and ticks suggesting the association of these genotypes to the ASFV sylvatic cycle as previously described [16,20,54]. The ASFV p72 genotype V was reported to circulate in Malawi and Mozambique since 1960 and the sylvatic cycle was cited to play an important role in its maintenance in those countries [20,49]. The genotype X was recovered from domestic pigs in 1950 in Kenya [15], since then, it was regularly reported to circulate and cause ASF outbreaks in Kenya [15,16,21,23], Uganda [21,23], Tanzania [22,23,61,62], Burundi [22,23,64], and DRC [51]. Recent molecular studies have reported high genetic similarity at three genomic regions of ASFV (p72, CVR and *I73R-I329L*) between the ASFV p72 genotype X strains responsible for ASF outbreaks in 2016 in Kagera region in Tanzania [62,64], the 2018 ASF outbreak in Rutana region in Burundi [64], and the ASF outbreak that occurred during December 2018 to January 2019 in South Kivu province of the DRC [51]. Commercial traffic and cross-border movements of domestic pigs and pork products were speculated as the main drivers of the ASFV spread between DRC, Burundi, and Tanzania [51,53,64]. Rutana region shares a border with Kagera region which borders South Kivu province through Lake Tanganyika and uncontrolled transboundary movements of domestic pigs and pork products are likely to happen in the area. However, the area is rich in wildlife protected areas with natural reservoir of ASFV (mainly warthogs) and their role in the epidemiology of the disease in the area has not been investigated. It would be interesting to investigate the sylvatic cycle of the ASFV in the area in order to get more insight into the epidemiology of ASFV. The included studies reported the ASFV p72 genotype I isolated from domestic pigs, ticks and bush pigs. Genotype I was described as the most prevalent in DRC where it was regularly recovered from domestic pigs since 1963 [22,23,53] and it is described as the most widely spread ASFV genotype in Zambia where it was isolated from soft ticks and domestic pigs [38,40,41]. Additionally, one study has described the ASFV p72 genotype I isolated from bush pig (*Potamochoerus porcus*) in 1961 in Kenya [22]. Apart from eastern and southern Africa, the ASFV p72 genotype I strains have a wide range of geographical distribution, they have been described in Europe, South America, the Caribbean, and West Africa [22].

The ASFV p72 genotype IX was recovered from domestic pigs and warthogs. This genotype is described as the most predominant in Uganda and Kenya [42], but also, it was described in DRC [50,53] and Tanzania [56,62]. Although associated with lethal ASF outbreaks [21,47,48], an increasing number of studies reported the genotype IX in asymptomatic domestic pigs in Tanzania [56], DRC [50], Uganda [44], and Kenya [65]. The reason behind this variation in virulence of the ASFV p72 genotype IX is worth investigating. The high genetic similarity between the ASFV p72 genotype IX strains recovered from warthogs in central Kenya in 2008 and 2009 [16] to ASFV strains described in domestic pigs in Kenya, Uganda, DRC, and Tanzania highlights the role of the ASFV sylvatic cycle in the epidemiology of the ASFV in eastern Africa.

The included studies reported ASFV p72 genotypes II, VIII, XII, XIV, and XV in both domestic pigs and ticks. The ASFV p72 genotype II was recovered from domestic pigs in Zambia and Mozambique about 30 years ago [22,49] and since then it has expended its geographical distribution not only to eastern and southern Africa countries but also to Madagascar [66], Mauritius [67], Europe [24], and Asia [11,25]. The recent detection of this genotype II in soft ticks in Mozambique provides evidence of a possible sylvatic source of this ASFV p72 genotype [20]. In addition, homology between ASFV strains from ticks collected at pig shelters and those from warthog burrows was observed, suggesting the possibility of ASFV transmission at the wild and domestic pigs interface [20]. Among countries included in this review, the ASFV p72 genotype II was described in Zambia [17,40], Mozambique [20,22,49], Tanzania [56,59,62], and in Malawi [63]. In 2007, the ASFV p72 genotype II escaped from its African geographical distribution to Georgia and subsequently spread to countries of the European Union and Russia before it reached Asia in August 2018 [13,25]. Additionally, the transboundary spread of the highly virulent ASFV p72 genotype II similar to the Georgia 2007/1 strain is highlighted by the introduction of this genotype into Tanzania probably from Malawi [59,62,63] and the emergence of this ASFV genotype in Zimbabwe in 2015 after several years of ASF absence probably from the neighboring Mozambique [68]. Furthermore, the genotype VIII was found to circulate in Zambia, Mozambique and Malawi [21,22,23], genotype XII was described in Zambia and Malawi [22], whilst genotype XV seems to be confined to Tanzania where both domestic and sylvatic cycles have been described for this genotype [22,57,58]. Additionally, genotype XIV which was previously restricted to Zambia where it was isolated from soft ticks in 1986 [22] was reported in domestic pigs in Zambia [17] and in DRC [53].

Genotypes VI and XVI were described as domestic pig cycle associated genotypes being confined to Mozambique [23,49] and Tanzania [22], respectively, while genotypes XI and XIII were recovered from soft ticks in Zambia in 1983 [22], and the recently identified genotype XXIV was isolated from soft ticks collected in 2006 from Gorongosa Park in Mozambique [20].

High genotypic variability was reported by included studies with 15 different ASFV genotypes being reported to circulate in countries concerned by this review. Movement of domestic pigs and pork products were cited as the main factor of ASFV spreading within and between countries, for instance the ASFV spread pattern was linked to trade highways in Tanzania [62] and Uganda [47] highlighting the importance of the anthropogenic factors and the ASFV domestic pig cycle in the spread of ASF in concerned countries. In addition, the ASFV sylvatic cycle seems to play a role in the maintenance of ASFV in concerned countries as 13 among the 15 circulating genotypes were isolated either from warthogs or ticks. However, the role of bush pigs in the epidemiology of ASF in the countries concerned by this review appears extremely limited as only one study reported ASFV genotype I recovered from bush pig.

This study identified substantial viral dispersal and spread routes between Tanzania and its neighboring countries. The estimated evolution rate of 4.805 × 10^−5^ substitution/site/year for ASFV strains collected in Tanzania and its eight neighboring countries from 1954 to 2019 is higher than other double-stranded DNA viruses, however, it is similar to the substitution rate of the rapidly evolving RNA viruses [69]. These findings are in agreement with previous studies that estimated the evolution rate for the ASFV strains [70,71]. Our estimated temporal most recent common ancestor (TMRCA) dated back to 1652.233 (1626.473, 1667.735) supporting the hypothesis that the ASFV could have been circulating in Eastern Africa before it was isolated and described for the first time as previously described [70,71]. Phylogeographical dispersal of the ASFV revealed several transboundary spread events from Kenya to Uganda, Tanzania and Mozambique. From Mozambique, ASFV further spread to Zambia and Malawi and from there to Tanzania. Transmissions from Uganda to DRC, from Tanzania to Burundi were also observed as shown in Figure 3. These observations are consistent with studies using traditional phylogenetic methods where transboundary spread of ASFV between DRC and Uganda [50,53], Tanzania and Malawi [59,63], DRC and Zambia [53], Kenya and Uganda [21] were speculated. Our findings highlight the role of neighboring countries in the epidemiology of ASFV and a regional approach would be more effective for the control of ASF. Whole genome sequences of ASFV from the countries concerned by this review are still limited and those data are needed for estimating more accurately the transmission dynamics of the ASFV in these countries and designing an effective control strategy.

## 5. Conclusions

In conclusion, a considerable diversity of ASFV genotypes were found to circulate in Tanzania and its neighboring countries. Furthermore, a transboundary spread of ASFV between countries was observed. These results suggest persistence of ASFV in these countries and advocate for more research on whole genome sequencing of ASFV and the ASFV sylvatic cycle to improve our understanding of the transmission dynamics of the virus and for a regional approach to mitigate the spread of ASFV.

## Figures and Tables

**Table 1:** African swine fever outbreaks, cases, deaths, and case fatality rate reported to the World Organization for Animal Health (OIE) by Tanzania and its eight neighboring countries from 2005 to 2019.

Time Period	Country	Number of Outbreaks	Number of Cases	Number of Deaths	Case Fatality Rate (%)
**2005–2009**	Tanzania	5	956	738	77.19
	Rwanda	134	7057	5863	83.08
	Burundi	-	-	-	-
	Malawi	86	16,973	10,785	63.54
	DRC	81	1413	1329	94.05
	Mozambique	78	6715	5194	77.35
	Zambia	43	1570	1271	80.95
	Kenya	9	924	549	59.41
	Uganda	3	401	181	45.13
**Subtotal**		439	36,009	25,910	71.95
**2010–2014**	Tanzania	41	4957	4275	86.24
	Rwanda	200	3553	1068	30.06
	Burundi	1	159	26	16.35
	Malawi	139	80,437	77,896	96.84
	DRC	191	153,692	140,493	91.41
	Mozambique	42	3136	2391	76.24
	Zambia	44	3835	2381	62.08
	Kenya	6	203	167	82.26
	Uganda	10	622	473	76.04
**Subtotal**		674	250,594	229,170	91.45
**2015–2019**	Tanzania	43	4981	3067	61.57
	Rwanda	47	593	532	89.71
	Burundi	28	3633	560	15.41
	Malawi	19	1813	1666	91.89
	DRC	237	35,407	35,038	98.95
	Mozambique	38	1239	936	75.54
	Zambia	42	5966	5025	84.23
	Kenya	3	231	223	96.53
	Uganda	18	1276	612	47.96
**Subtotal**		475	55,139	47,659	86.43
**Grand total**		1588	341,742	302,739	88.58

**Table 2:** Summary of the ASFV p72 genotypes distribution in Tanzania and neighboring countries. The black highlight indicates that the corresponding genotype is present.

	ASFV p72 Genotype															
**Country**	**I**	**II**	**V**	**VI**	**VIII**	**IX**	**X**	**XI**	**XII**	**XIII**	**XIV**	**XV**	**XVI**	**XX**	**XXIV**	**Total** **Genotypes**
Burundi																1
DRC																5
Kenya																3
Malawi																4
Mozambique																5
Rwanda																0
Tanzania																5
Uganda																2
Zambia																7
Total countries	3	4	2	1	3	4	5	1	2	1	2	1	1	1	1	

**Table 3:** African swine fever virus p72 genotypes circulating between Tanzania and its eight neighboring countries by October 2020.

Reference	Targeted Genomic Region	Reported ASFV p72 Genotype	Host Species	Country (Number of Papers)
[38]	p72	I	Tick	Zambia (7)
[39]	Whole genome sequencing	I	Tick	
[40]	p72, p54, p30, CVR	II	Domestic pig	
[41]	p72, p54	I	Domestic pig	
[22]	p72	I, II, VIII, XI, XII, XIII, XIV	Domestic pig and tick	
[23]	p72, CVR	I, VIII	Tick and domestic pig	
[17]	p72, p54, CVR	I, II, XIV	Domestic pig	
[42]	WGS	IX	Domestic pig	Uganda (10)
[43]	p72, p54, CVR	IX	Domestic pig	
[44]	p72, p54, CVR	IX	Domestic pig	
[45]	p72, p54, CVR	IX	Domestic pig	
[46]	WGS	IX	Domestic pig	
[47]	p72, p54, CVR	IX	Domestic pig	
[21]	p72, p54, CVR	IX, X	Domestic pig	
[22]	p72	IX	Domestic pig	
[23]	p72, CVR	IX, X	Domestic pig	
[48]	p72, p54, CVR, TK	IX	Domestic pig	
[49]	p72, CVR	II, V, VI, VIII	Domestic pig	Mozambique (5)
[21]	p72, p54, CVR	V	Domestic pig	
[22]	p72	II, VIII	Domestic pig	
[23]	p72, CVR	VI	Domestic pig	
[20]	p72, CVR, p30, p54	II, V, XXIV	Tick	
[50]	p72, p54	IX	Domestic pig	DRC (6)
[51]	p72, p54, CVR, CD2v, I73R-I329L	X	Domestic pig	
[52]	Whole genome sequencing	XX	Domestic pig	
[22]	p72	I	Not known	
[53]	p72, p54, CVR	I, IX, XIV	Domestic pig	
[23]	p72, CVR	I	Domestic pig	
[54]	WGS	IX, X	Domestic pig	Kenya (8)
[15]	WGS	X	Domestic pig	
[16]	p72, p54, p30, CVR	IX, X	Domestic pig, tick and warthog	
[21]	p72, p54, CVR	IX	Domestic pig	
[48]	p72, p54, CVR, TK	IX	Domestic pig	
[55]	p72, p54	IX	Domestic pig	
[22]	p72	I, X	Warthog, domestic pig and bush pig	
[23]	p72, CVR	X	Warthog	
[56]	p72, p54, CVR, I73R-I329L	II, IX	Domestic pig	Tanzania (9)
[57]	p72, p54, CVR	XV	Tick	
[58]	p72, p54, CVR	XV	Domestic pig	
[59]	p72	II	Domestic pig	
[60]	p72, p54, CVR	X	Domestic pig	
[61]	p72	X	Domestic pig	
[22]	p72	X, XV, XVI	Tick, warthog and domestic pig	
[23]	p72, CVR	X	Warthog	
[62]	p72	II, IX, X	Domestic pig	
[63]	p72, CVR, I73R-I329L	II	Domestic pig	Malawi (4)
[21]	p72, p54, CVR	V, VIII	Domestic pig and tick	
[22]	p72	VIII, V, XII	Domestic pig	
[23]	p72, CVR	V, VIII	Warthog and domestic pig	
[22]	p72	X	Domestic pig	Burundi (3)
[23]	p72, CVR	X	Domestic pig	
[64]	p72, CVR, I73R-I329L	X	Domestic pig	
**-**	-	-	-	Rwanda (0)

## References

[1] Costard S., Zagmutt F.J., Porphyre T., Pfeiffer D.U. (2015). Small-scale pig farmers’ behavior, silent release of African swine fever virus and consequences for disease spread. Sci. Rep..

[2] Montgomery E.R. (1921). On a form of swine fever occurring in British East Africa (Kenya Colony). J. Comp. Pathol. Ther..

[3] Fasina F.O., Kissinga H., Mlowe F., Mshang’a S., Matogo B., Mrema A., Mhagama A., Makungu S., Mtui-Malamsha N., Sallu R. (2020). Drivers, risk factors and dynamics of African Swine fever outbreaks, Southern Highlands, Tanzania. Pathogens.

[4] Mulumba-Mfumu L.K., Saegerman C., Dixon L.K., Madimba K.C., Kazadi E., Mukalakata N.T., Oura C.A.L., Chenais E., Masembe C., Ståhl K. (2019). African swine fever: Update on Eastern, central and Southern Africa. Transbound Emerg. Dis..

[5] Penrith M.-L., Bastos A.D., Etter E.M.C., Beltrán-Alcrudo D. (2019). Epidemiology of African swine fever in Africa today: Sylvatic cycle versus socio-economic imperatives. Transbound Emerg. Dis..

[6] Costard S., Wieland B., de Glanville W., Jori F., Rowlands R., Vosloo W., Roger F., Pfeiffer D.U., Dixon L.K. (2009). African swine fever: How can global spread be prevented?. Philos. Trans. R Soc. B Biol. Sci..

[7] Loi F., Cappai S., Coccollone A., Rolesu S. (2019). Standardized risk analysis approach aimed to evaluate the last African swine fever eradication program performance, in Sardinia. Front. Vet. Sci..

[8] Penrith M.-L. (2013). History of “swine fever” in southern Africa. J. S. Afr. Vet. Assoc..

[9] Li X., Tian K. (2018). African swine fever in China. Vet. Rec..

[10] OIE (2020). World Animal Health Information System. http://www.oie.int/wahis_2/public/wahid.php/Countryinformation/Reporting.

[11] Kim H.-J., Cho K.-H., Ryu J.-H., Jang M.-K., Chae H.-G., Choi J.-D., Nah J.-J., Kim Y.-J., Kang H.-E. (2020). Isolation and genetic characterization of African Swine fever virus from domestic pig farms in South Korea, 2019. Viruses.

[12] Vergne T., Chen-Fu C., Li S., Cappelle J., Edwards J., Martin V., Pfeiffer D.U., Fusheng G., Roger F.L. (2017). Pig empire under infectious threat: Risk of African swine fever introduction into the People’s Republic of China. Vet. Rec..

[13] Rowlands R.J., Michaud V., Heath L., Hutchings G., Oura C., Vosloo W., Dwarka R., Onashvili T., Albina E., Linda K. (2008). African swine fever Virus Isolate, Georgia, 2007. Emerg. Infect. Dis..

[14] Alonso C., Borca M., Dixon L., Revilla Y., Rodriguez F., Escribano J.M., ICTV Report Consortium (2018). ICTV virus taxonomy profile: Asfarviridae. J. Gen. Virol..

[15] de Villiers E.P., Gallardo C., Arias M., da Silva M., Upton C., Martin R., Bishop R.P. (2010). Phylogenomic analysis of 11 complete African swine fever virus genome sequences. Virology.

[16] Gallardo C., Okoth E., Pelayo V., Anchuelo R., Martin E., Simon A., Llorente A., Nieto R., Soler A., Martín R. (2011). African swine fever viruses with two different genotypes, both of which occur in domestic pigs, are associated with ticks and adult warthogs, respectively, at a single geographical site. J. Gen. Virol..

[17] Simulundu E., Chambaro H.M., Sinkala Y., Kajihara M., Ogawa H., Mori A., Ndebe J., Dautu G., Mataa L., Lubaba C.H. (2017). Co-circulation of multiple genotypes of African swine fever viruses among domestic pigs in Zambia (2013–2015). Transbound Emerg. Dis..

[18] Bastos A.D.S., Penrith M.-L., Crucière C., Edrich J.L., Hutchings G., Roger F., Couacy-Hymann E., Thomson R.G. (2003). Genotyping field strains of African swine fever virus by partial p72 gene characterisation. Arch. Virol..

[19] Achenbach J.E., Gallardo C., Nieto-Pelegrín E., Rivera-Arroyo B., Degefa-Negi T., Arias M., Jenberie S., Mulisa D.D., Gizaw D., Gelaye E. (2017). Identification of a new genotype of African Swine fever virus in domestic pigs from ethiopia. Transbound Emerg. Dis..

[20] Quembo C.J., Jori F., Vosloo W., Heath L. (2018). Genetic characterization of African swine fever virus isolates from soft ticks at the wildlife/domestic interface in Mozambique and identification of a novel genotype. Transbound Emerg. Dis..

[21] Gallardo C., Mwaengo D.M., Macharia J.M., Arias M., Taracha E.A., Soler A., Okoth E., Martín E., Kasiti J., Bishop R.P. (2009). Enhanced discrimination of African swine fever virus isolates through nucleotide sequencing of the p54, p72, and pB602L (CVR) genes. Virus Genes.

[22] Lubisi B.A., Bastos A.D.S., Dwarka R.M., Vosloo W. (2005). Molecular epidemiology of African swine fever in East Africa. Arch. Virol..

[23] Nix R.J., Gallardo C., Hutchings G., Blanco E., Dixon L.K. (2006). Molecular epidemiology of African swine fever virus studied by analysis of four variable genome regions. Arch. Virol..

[24] Gallardo C., Fernández-Pinero J., Pelayo V., Gazaev I., Markowska-Daniel I., Pridotkas G., Nieto R., Fernández-Pacheco P., Bokhan S., Nevolko O. (2014). Genetic variation among African Swine fever genotype II viruses, eastern and central Europe. Emerg. Infect. Dis..

[25] Ge S., Li J., Fan X., Liu F., Li L., Wang Q., Ren W., Bao J., Liu C., Wang H. (2018). Molecular characterization of African swine fever virus, China, 2018. Emerg. Infect. Dis..

[26] Sanna G., Dei Giudici S., Bacciu D., Angioi P.P., Giammarioli M., De Mia G.M., Oggiano A. (2017). Improved strategy for molecular characterization of African Swine fever viruses from Sardinia, based on analysis of p30, CD2V and I73R/I329L variable regions. Transbound Emerg. Dis..

[27] Chenais E., Ståhl K., Guberti V., Depner K. (2018). Identification of Wild Boar–Habitat Epidemiologic Cycle in African Swine Fever Epizootic-Volume 24, Number 4—April 2018-Emerging Infectious Diseases Journal-CDC. https://wwwnc.cdc.gov/eid/article/24/4/17-2127_article.

[28] Moher D., Liberati A., Tetzlaff J., Altman D.G., Group T.P. (2009). Preferred reporting items for systematic reviews and meta-analyses: The PRISMA statement. PLoS Med..

[29] Ouzzani M., Hammady H., Fedorowicz Z., Elmagarmid A. (2016). Rayyan—A web and mobile app for systematic reviews. Syst. Rev..

[30] Harzing A.-W. (2007). Publish or Perish. Harzing.com. https://harzing.com/resources/publish-or-perish.

[31] Kumar S., Stecher G., Li M., Knyaz C., Tamura K. (2018). MEGA X: Molecular evolutionary genetics analysis across computing platforms. Mol. Biol. Evol..

[32] Gouy M., Guindon S., Gascuel O. (2010). Sea view version 4: A multiplatform graphical user interface for sequence alignment and phylogenetic tree building. Mol. Biol. Evol..

[33] Lefort V., Longueville J.-E., Gascuel O. (2017). SMS: Smart model selection in PhyML. Mol. Biol. Evol..

[34] Rambaut A., Lam T.T., Max Carvalho L., Pybus O.G. (2016). Exploring the temporal structure of heterochronous sequences using TempEst (formerly Path-O-Gen). Virus Evol [Internet]. https://academic.oup.com/ve/article/2/1/vew007/1753488.

[35] To T.-H., Jung M., Lycett S., Gascuel O. (2016). Fast dating using least-squares criteria and algorithms. Syst. Biol..

[36] Ishikawa S.A., Zhukova A., Iwasaki W., Gascuel O. (2019). A fast likelihood method to reconstruct and visualize ancestral scenarios. Mol. Biol. Evol..

[37] Letunic I., Bork P. (2019). Interactive Tree Of Life (iTOL) v4: Recent updates and new developments. Nucleic Acids Res..

[38] Chambaro H.M., Sasaki M., Sinkala Y., Gonzalez G., Squarre D., Fandamu P., Lubaba C., Mataa L., Shawa M., Mwape K.E. (2020). Evidence for exposure of asymptomatic domestic pigs to African swine fever virus during an inter-epidemic period in Zambia. Transbound Emerg. Dis..

[39] Ndlovu S., Williamson A.-L., Malesa R., Heerden J van Boshoff C.I., Bastos A.D.S., Heath L., Carulei O. (2020). Genome sequences of three African Swine fever viruses of genotypes I, III, and XXII from South Africa and Zambia, isolated from Ornithodoros Soft Ticks. Microbiol. Resour. Announc..

[40] Simulundu E., Sinkala Y., Chambaro H.M., Chinyemba A., Banda F., Mooya L.E., Ndebe J., Chitanga S., Makungu C., Munthali G. (2018). Genetic characterisation of African swine fever virus from 2017 outbreaks in Zambia: Identification of p72 genotype II variants in domestic pigs. Onderstepoort J. Vet. Res..

[41] Thoromo J., Simulundu E., Chambaro H.M., Mataa L., Lubaba C.H., Pandey G.S., Takada A., Misinzo G., Mweene A.S. (2016). Diagnosis and genotyping of African swine fever viruses from 2015 outbreaks in Zambia. Onderstepoort J. Vet. Res..

[42] Filipe A.D.S., Vattipally S.B., Mair D., Ogweng P., Mayega J., Muwanika V., Palmarini M., Biek R., Masembe C. (2019). Host genome depletion to determine the evolution, genetic diversity and transmission patterns of full genome sequences of African swine fever genotype IX from Uganda. Access Microbiol. Microbiol. Soc..

[43] Atuhaire D.K., Afayoa M., Ochwo S., Mwesigwa S., Okuni J.B., Olaho-Mukani W., Ojok L. (2013). Molecular characterization and phylogenetic study of African swine fever virus isolates from recent outbreaks in Uganda (2010–2013). Virol. J..

[44] Atuhaire D.K., Ochwo S., Afayoa M., Mwesigwa S., Mwiine F.N., Okuni J.B., Olaho-Mukani W., Ojok L. (2014). Molecular characterization of African swine fever virus in apparently healthy domestic pigs in Uganda. Afr. J. Biotechnol..

[45] Gallardo C., Ademun A.R., Nieto R., Nantima N., Arias M., Martín E., Pelayo V., Bishop R.P. (2011). Genotyping of African swine fever virus (ASFV) isolates associated with disease outbreaks in Uganda in 2007. Afr. J. Biotechnol..

[46] Masembe C., Sreenu V.B., Filipe A.D.S., Wilkie G.S., Ogweng P., Mayega F.J., Muwanika V.B., Biek R., Palmarini M., Davison A.J. (2018). Genome sequences of five African Swine fever virus genotype IX isolates from domestic pigs in Uganda. Microbiol. Resour. Announc..

[47] Mwiine F.N., Nkamwesiga J., Ndekezi C., Ochwo S. (2019). Molecular characterization of African Swine fever viruses from outbreaks in Peri-Urban Kampala, Uganda. Adv. Virol..

[48] Onzere C.K., Bastos A.D., Okoth E.A., Lichoti J.K., Bochere E.N., Owido M.G., Ndambuki G., Bronsvoort M., Bishop R.P. (2018). Multi-locus sequence typing of African swine fever viruses from endemic regions of Kenya and Eastern Uganda (2011–2013) reveals rapid B602L central variable region evolution. Virus Genes..

[49] Bastos A.D.S., Penrith M.-L., Macome F., Pinto F., Thomson G.R. (2004). Co-circulation of two genetically distinct viruses in an outbreak of African swine fever in Mozambique: No evidence for individual co-infection. Vet. Microbiol..

[50] Bisimwa P.N., Machuka E.M., Githae D., Banswe G., Amimo J.O., Ongus J.R., Masembe C., Bishop R.P., Steinaa L., Djikeng A. (2020). Evidence for the presence of African swine fever virus in apparently healthy pigs in South-Kivu Province of the Democratic Republic of Congo. Vet. Microbiol..

[51] Bisimwa P.N., Ongus J.R., Tiambo C.K., Machuka E.M., Bisimwa E.B., Steinaa L., Pelle R. (2020). First detection of African swine fever (ASF) virus genotype X and serogroup 7 in symptomatic pigs in the Democratic Republic of Congo. Virol. J..

[52] Ndlovu S., Williamson A., Heath L., Carulei O. (2020). Genome sequences of three African Swine fever viruses of genotypes IV and XX from Zaire and South Africa, isolated from a domestic Pig (Sus scrofa domesticus), a Warthog (Phacochoerus africanus), and a European Wild Boar (Sus scrofa). Microbiol. Resour. Announc..

[53] Mulumba–Mfumu L.K., Achenbach J.E., Mauldin M.R., Dixon L.K., Tshilenge C.G., Thiry E., Moreno N., Blanco E., Saegerman C., Lamien C.E. (2017). Genetic assessment of African Swine fever isolates involved in outbreaks in the democratic Republic of Congo between 2005 and 2012 reveals Co-circulation of p72 genotypes I, IX and XIV, including 19 variants. Viruses.

[54] Bishop R.P., Fleischauer C., de Villiers E.P., Okoth E.A., Arias M., Gallardo C., Upton C. (2015). Comparative analysis of the complete genome sequences of Kenyan African swine fever virus isolates within p72 genotypes IX and X. Virus Genes.

[55] Thomas L.F., Bishop R.P., Onzere C., Mcintosh M.T., Lemire K.A., de Glanville W.A., Cook E.A.J., Fèvre E.M. (2016). Evidence for the presence of African swine fever virus in an endemic region of Western Kenya in the absence of any reported outbreak. BMC Vet. Res..

[56] Chang’a J.S., Mayenga C., Settypalli T.B.K., Achenbach J.E., Mwanandota J.J., Magidanga B., Cattoli G., Jeremiah M., Kamigwe A., Guo S. (2019). Symptomatic and asymptomatic cases of African swine fever in Tanzania. Transbound Emerg. Dis..

[57] Peter E., Machuka E., Githae D., Okoth E., Cleaveland S., Shirima G., Kusiluka L., Pelle R. (2020). Detection of African swine fever virus genotype XV in a sylvatic cycle in Saadani National Park, Tanzania. Transbound Emerg. Dis..

[58] Misinzo G., Magambo J., Masambu J., Yongolo M.G., Van Doorsselaere J., Nauwynck H.J. (2011). Genetic characterization of African swine fever viruses from a 2008 outbreak in Tanzania. Transbound Emerg. Dis..

[59] Misinzo G., Kasanga C.J., Mpelumbe–Ngeleja C., Masambu J., Kitambi A., Van Doorsselaere J. (2012). African Swine fever virus, Tanzania, 2010–2012. Emerg. Infect. Dis..

[60] Misinzo G., Kwavi D.E., Sikombe C.D., Makange M., Peter E., Muhairwa A.P., Madege M.J. (2014). Molecular characterization of African swine fever virus from domestic pigs in northern Tanzania during an outbreak in 2013. Trop. Anim. Health Prod..

[61] Misinzo G., Jumapili F., Ludosha M., Mafie E., Silialis J., Mushi R., Viaene W., Doorsselaere J.V. (2012). Genotyping of African swine fever virus from a 2009 outbreak in Tanzania. Res. Opin. Anim. Vet. Sci..

[62] Yona C.M., Vanhee M., Simulundu E., Makange M., Nauwynck H.J., Misinzo G. (2020). Persistent domestic circulation of African swine fever virus in Tanzania, 2015–2017. BMC Vet. Res..

[63] Hakizimana J.N., Kamwendo G., Chulu J.L.C., Kamana O., Nauwynck H.J., Misinzo G. (2020). Genetic profile of African swine fever virus responsible for the 2019 outbreak in northern Malawi. BMC Vet. Res..

[64] Hakizimana J.N., Nyabongo L., Ntirandekura J.B., Yona C., Ntakirutimana D., Kamana O., Nauwynck H., Misinzo G. (2020). Genetic analysis of African Swine fever virus from the 2018 outbreak in South-Eastern Burundi. Front. Vet. Sci..

[65] Abworo E.O., Onzere C., Oluoch Amimo J., Riitho V., Mwangi W., Davies J., Blome S., Bishop R.P. (2017). Detection of African swine fever virus in the tissues of asymptomatic pigs in smallholder farming systems along the Kenya-Uganda border: Implications for transmission in endemic areas and ASF surveillance in East Africa. J. Gen. Virol..

[66] Gonzague M., Roger F., Bastos A., Burger C., Randriamparany T., Smondack S., Cruciere C. (2001). Isolation of a non-haemadsorbing, non-cytopathic strain of African swine fever virus in Madagascar. Epidemiol. Infect..

[67] Lubisi B.A., Dwarka R.M., Meenowa D., Jaumally R. (2009). An investigation into the first outbreak of African swine fever in the Republic of Mauritius. Transbound Emerg. Dis..

[68] van Heerden J., Malan K., Gadaga B.M., Spargo R.M. (2017). Reemergence of African Swine Fever in Zimbabwe, 2015. Emerg. Infect. Dis..

[69] Duffy S., Shackelton L.A., Holmes E.C. (2008). Rates of evolutionary change in viruses: Patterns and determinants. Nat. Rev. Genet..

[70] Alkhamis M.A., Gallardo C., Jurado C., Soler A., Arias M., Sánchez-Vizcaíno J.M. (2018). Phylodynamics and evolutionary epidemiology of African swine fever p72-CVR genes in Eurasia and Africa. PLoS ONE.

[71] Michaud V., Randriamparany T., Albina E. (2013). Comprehensive phylogenetic reconstructions of African Swine fever virus: Proposal for a new classification and molecular dating of the Virus. PLoS ONE.

